# Research trajectory and future trends in curcumin related to immunity: a bibliometric analysis of publications from last two decades

**DOI:** 10.3389/fimmu.2025.1559670

**Published:** 2025-03-24

**Authors:** Qing Hong, Wei Lyu, Chaowei Zhang, Weiyi Yao, Yuxuan Han, Na Chen

**Affiliations:** ^1^ Department of Hematology, Shandong Provincial Hospital Affiliated to Shandong First Medical University, Jinan, China; ^2^ School of Economics and Management, Anhui Polytechnic University, Wuhu, China

**Keywords:** curcumin, immunity, bibliometric analysis, Citespace, anti-cancer

## Abstract

Curcumin has a clear immunopharmacological effect and plays an important role as an immune agent in various immune diseases and tumor immunotherapy. To comprehensively and scientifically clarify and reflect the development process, current status, and research trends of curcumin in the field of immune regulation, and to provide reliable insights for discipline development strategies and future research expansion, this study systematically analyzes 3939 valid articles related to curcumin and immunity published between 2004 and 2024 from the Web of Science database. Using Citespace and R-bibliometrix software for bibliometric analysis, we create visual knowledge maps from multiple dimensions including overall publication output, influential research entities, highly cited papers, research topics and hotspots. The results indicate that the overall number of publications and citations is currently in a rapid development phase. China occupies a core position in this research field but has low collaboration intensity. The Egyptian Knowledge Bank (EKB) is the institution with the highest publication volume. Moreover, cluster analysis reveals that research hotspots are gradually shifting from fundamental pathology to topics involving broad social and environmental influences. The top five keywords with the most explosive citations—curcumin, inflammation, apoptosis, oxidative stress, and cancer—represent the most focused and influential research topics. Currently, curcumin immunology has developed a diversified research perspective, accumulating significant research in the areas of active substance basis, pharmacological activity, anti-inflammatory, and anti-cancer studies. The thematic evolution trends and keywords related to curcumin’s immunological mechanisms summarized in this article provide insights and guidance for future research directions.

## 1 Introduction

Curcumin is a natural spice extracted from the rhizomes of plants in the ginger family and the arum family (such as turmeric and calamus) (1). It contains an unsaturated fatty group backbone and aromatic structure, with a molecular weight of 368.37, and is classified as an acidic polyphenolic natural organic compound (2). Curcumin is the most abundant chemical component in the medicinal plant turmeric and can be processed for medicinal uses (3). It currently plays a vital role in cancer and cardiovascular disease prevention (4), liver protection with increased bile secretion, and inhibition of bacterial propagation (5), as well as suppression of inflammatory response diffusion in the human body (6, 7). Curcumin can regulate multiple immune cells (including B lymphocytes, T lymphocytes, dendritic cells, monocytes-macrophages, natural killer cells, and neutrophils) (8, 9)and cytokines (10), participating in various immune response processes (such as humoral immunity, cellular immunity, and autoimmunity) to reduce inflammatory responses while enhancing immune cell recognition, presentation, and killing of tumor cells (11, 12). Modern studies have applied curcumin in treating autoimmune diseases (13), various types of tumors, HIV, hepatitis B-related liver cirrhosis, and even the novel coronavirus (14), achieving promising progress (15). As the mechanisms of curcumin in cellular immunity are further elucidated, its clinical effects are broadly confirmed, with expectations for greater therapeutic roles in immune-related diseases in the future (16, 17).

In order to clarify the development context, current status, and evolving trends of curcumin’s immune function research while avoiding the limitations and influences of subjective factors in traditional review reading, this study uses bibliometric methods based on the perspective of scientific output. We utilized the SCI/SSCI papers of the Web of Science (WoS) database as the data sample basis for a multi-dimensional and temporal dynamic analysis and processing of literature characteristics, focusing on overall output, research entities and cooperative networks, highly cited literature, keyword co-occurrence, and burst keyword analyses. Visual scientific knowledge maps are used for an intuitive and clear display, aiming to comprehensively, scientifically, and multilaterally detect and reflect the research status, changing patterns, and dynamic evolution trends of curcumin’s immune function knowledge domain, providing references and insights for discipline development and future research expansion.

## 2 Materials and methods

### 2.1 Search strategy

Using the Web of Science database, we retrieved literature related to curcumin and immunity from 2004 to 2024, with a total of 3939 valid articles included for further analysis.

search database: WoS core collection.
search term: TS = ((immun*) AND (Curcumin OR Curcuma)).
inclusion criteria : Language = Enlglish.
document type = article or review.
publication year = 2004-2024.

### 2.2 Analysis tools

R-bibliometrix is an R package designed for scientific citation network analysis and bibliometric studies. It provides comprehensive tools for bibliographic data analysis and visualization, enabling complex citation network analyses such as citation coupling, co-citation, and collaboration analysis, helping to reveal interactions and influence relationships in scientific research. It employs techniques such as co-word analysis and scientific mapping to identify research trends and thematic evolution, offering various visualization tools, including scientific maps, keyword co-occurrence networks, and thematic evolution graphs.

CiteSpace, developed by Professor Chaomei Chen of Drexel University, is a bibliometric analysis and visualization software that runs on the Java platform. It aims to help researchers identify and understand trends and patterns within scientific literature, particularly excels at revealing the knowledge foundation and research frontiers of a specific research field.

## 3 Results

### 3.1 Publication and citation trend

**Figure 1:**
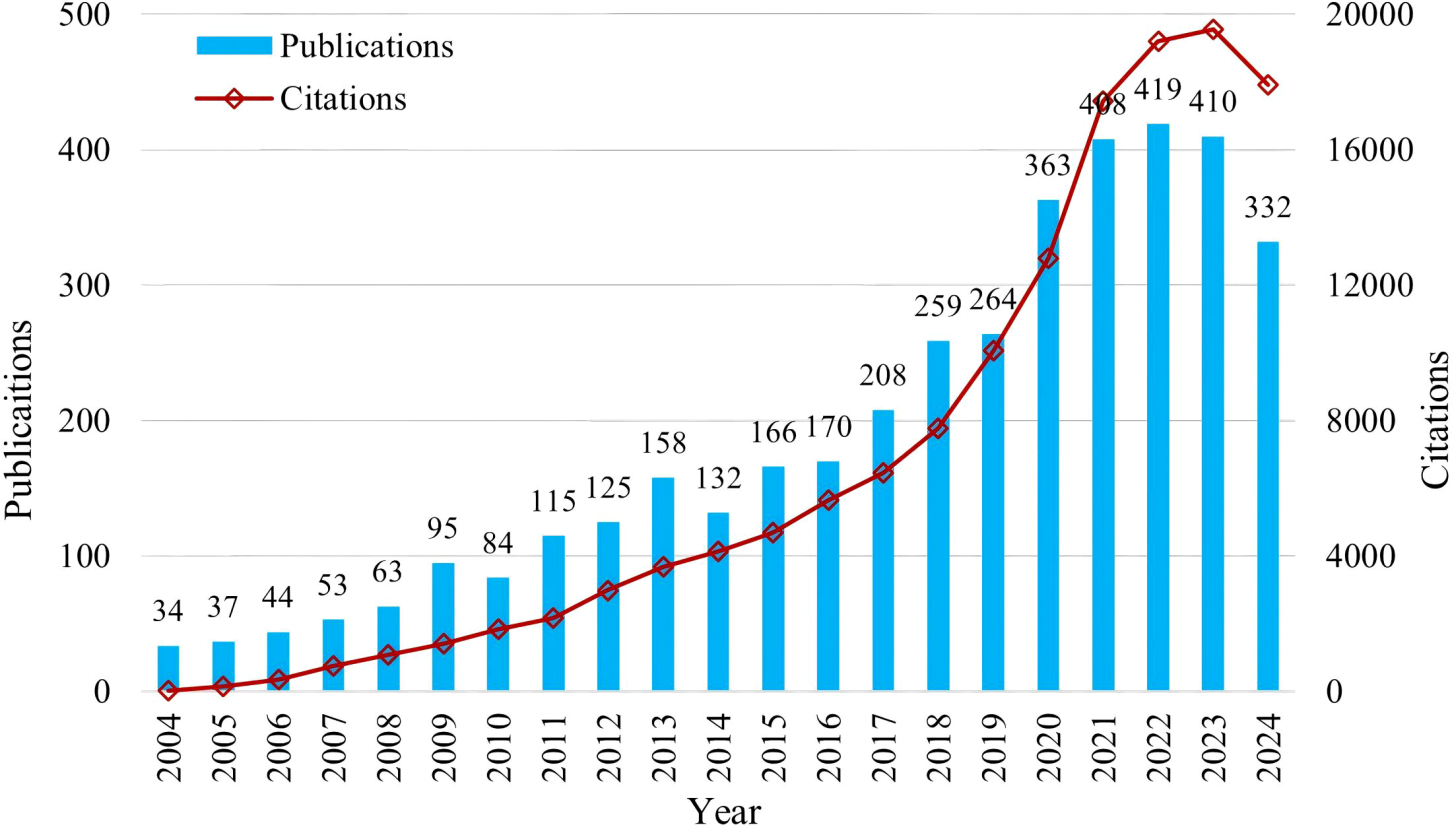
Publication output time distribution chart.

The distribution of literature quantity over time can reflect the research scale, development level, speed, and attention in this field. This allows for assessing the current research stage and predicting future trends—an essential indicator for measuring the development of a discipline. From the time distribution diagram of publication amounts shown in **Figure 1**, it can be observed that over the past 20 years, both the publication volume and citation volume in this field exhibit an exponential increase. After 20 years of ongoing exploration, demonstration, and development, a relatively stable and mature research system has formed in this discipline, laying a solid foundation for further in-depth studies. In recent years, the global outbreak of the COVID-19 pandemic led to the highest increase in publication volume in 2020, nearing 100 articles, and maintaining a stable level of high publication frequency in 2020 and 2023. Its publication and citation amounts show a significant leap compared to the previous decade, coinciding with the current pandemic timeline. This suggests that the research and role of curcumin in relation to COVID-19 are receiving significant attention. With advancements in research technology and the discovery of new breakthroughs, the publication volume on curcumin is expected to continue on a steady upward trend, although situations similar to the publication spike in 2020 may require specific timing.

### 3.2 Country performances

#### 3.2.1 Country publication volume

**Figure 2:**
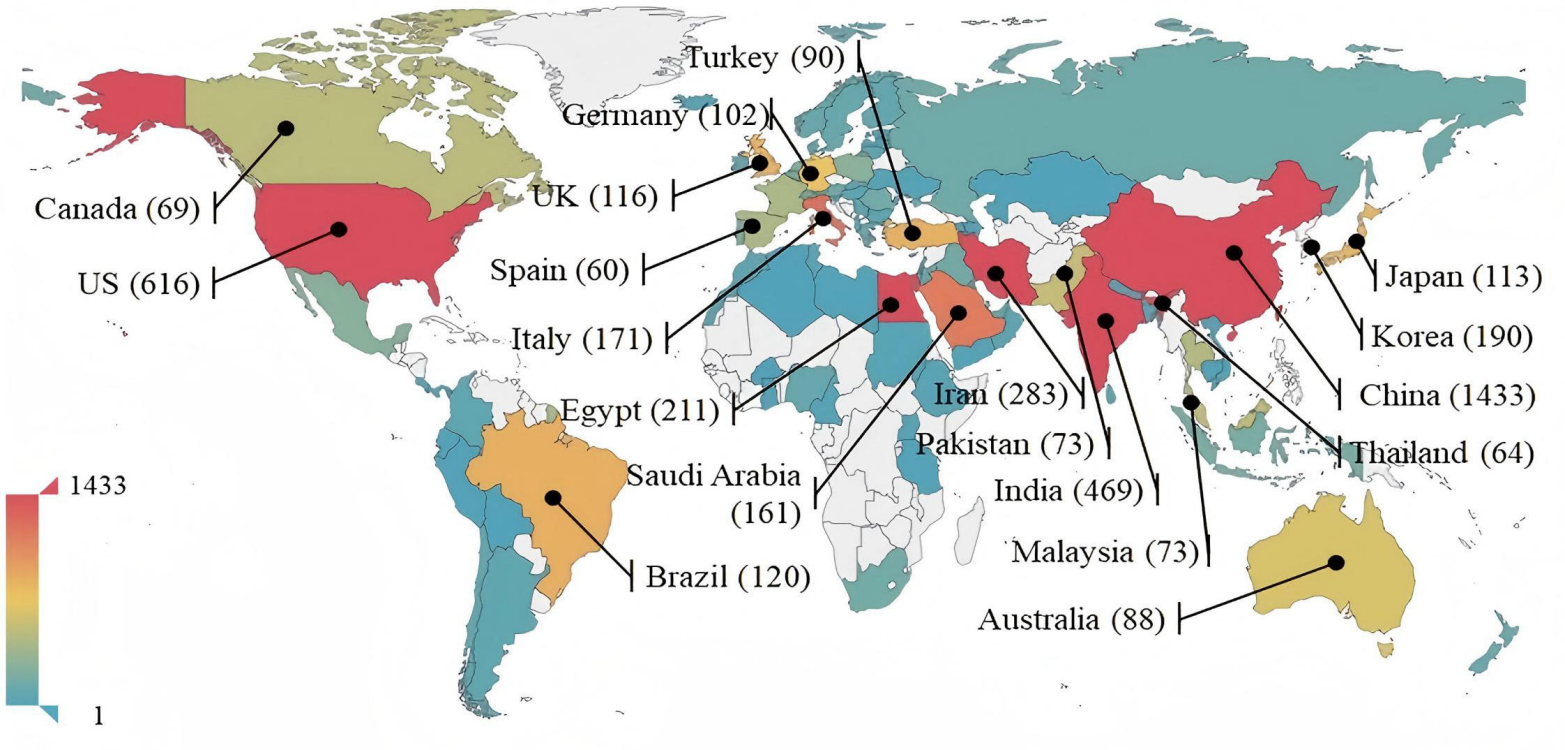
Corresponding countries’ publication volume.

From the search results, a total of 97 countries/regions contributed to the publications. In **Figure 2**, the countries with a publication volume exceeding 60 are presented, where China tops the list with 1433 publications, accounting for 33.38% of the total, indicating a significant advantage over other countries and regions, confirming China’s core position in this research field, likely due to curcumin being a component of traditional Chinese medicine and the increased focus on it in recent years. The United States ranks second with 616 publications, while India follows with 469 publications. The top 10 countries collectively account for over 90% of publications in this area. Among them, Asian countries represent half of the contributors, including China, India, Iran, South Korea, and Japan, indicating that other regions outside Asia have not yet gained sufficient attention in this field.

#### 3.2.2 Number of national cooperation publications

**Figure 3:**
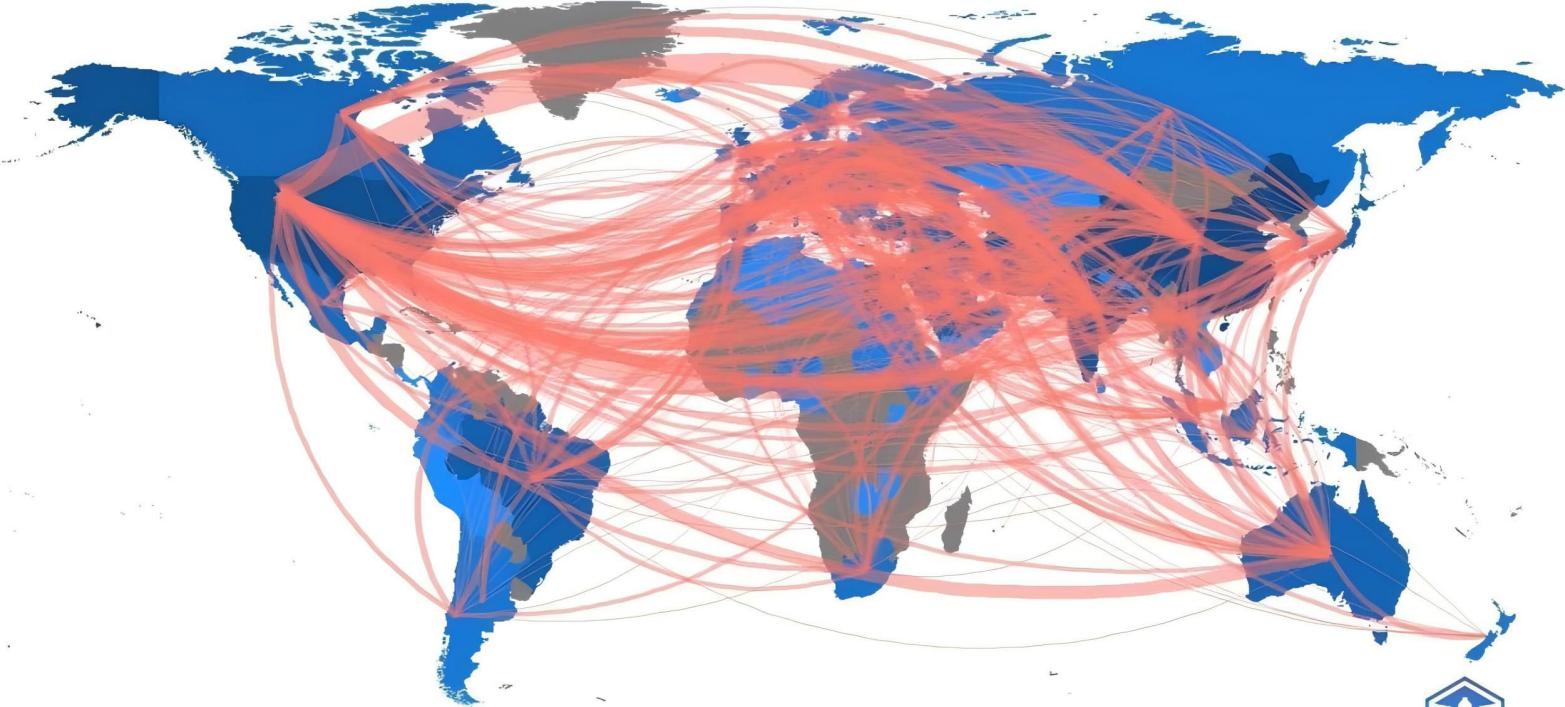
Country and region cooperation network graph.

In the cooperation network chart of countries shown in **Figure 3**, the node size represents the total publication output of that particular area in international collaboration, with line thickness indicating the relative strength of cooperation between connected countries and regions. Larger nodes and thicker lines reflect more active collaboration in the research of this field. Notably, China and the USA, as the top two countries in publication volume, have formed a significant cooperation network. The USA plays a central role in collaborative research on curcumin’s immune functions, engaging in extensive collaboration with China, India, and several Asian countries, as indicated in **Table 1**. Although China has a clear advantage in total research output, its collaborative relations with other countries are weaker than those of the USA. Overall, cooperation between countries/regions still remains relatively weak; the total number of collaborative papers between China and the USA, for instance, stands at only 110. This reflects that although a new international collaborative pattern is gradually forming, collaboration intensity remains low, and it has not yet become a primary mode of scientific research in this field. First, differences in the understanding of traditional medicine and modern medicine between different countries and regions may lead to a lack of willingness to cooperate. The mainstream scientific community in the West may be more inclined to rely on modern medicine, resulting in a lower acceptance of traditional Chinese medicine. The differences in research methodologies for traditional Chinese medicine affect the sharing and comparison of research outcomes. Second, there is a significant gap in funding and resource support between developed and developing countries, and there is a relative lack of international research platforms focused on curcumin and other traditional Chinese medicines. Therefore, enhancing international cooperation requires efforts across multiple levels, including cultural understanding, research standards, intellectual property protection, funding channels, and the formulation of policies and regulations, in order to promote in-depth research on traditional Chinese medicines like curcumin.

**Table 1:** National cooperation publication volume.

Country1	Country2	Freq	Country1	Country2	Freq
CHINA	USA	110	USA	GERMANY	12
EGYPT	SAUDI ARABIA	80	CHINA	EGYPT	11
USA	INDIA	62	CHINA	KOREA	11
USA	IRAN	37	CHINA	UNITED KINGDOM	11
INDIA	SAUDI ARABIA	35	INDIA	GERMANY	11
USA	AUSTRALIA	21	INDIA	KOREA	11
USA	ITALY	21	INDIA	MALAYSIA	11
USA	KOREA	21	CHINA	SAUDI ARABIA	10
IRAN	INDIA	18	ITALY	GERMANY	10
IRAN	UNITED KINGDOM	18	ITALY	PAKISTAN	10
SAUDI ARABIA	PAKISTAN	18	GERMANY	UNITED KINGDOM	9
IRAN	AUSTRALIA	17	INDIA	ITALY	9
CHINA	PAKISTAN	16	INDIA	PAKISTAN	9
CHINA	AUSTRALIA	15	IRAN	PAKISTAN	9
CHINA	JAPAN	15	SAUDI ARABIA	MALAYSIA	9
IRAN	ITALY	15	CHINA	GERMANY	8
USA	BRAZIL	15	CHINA	IRAN	8
USA	JAPAN	15	IRAN	IRAQ	8
USA	SAUDI ARABIA	15	ITALY	SAUDI ARABIA	8
USA	CANADA	14	ITALY	UNITED KINGDOM	8
USA	EGYPT	14	PAKISTAN	MALAYSIA	8
USA	UNITED KINGDOM	13	AUSTRALIA	UNITED KINGDOM	7
CHINA	CANADA	12	CHINA	FRANCE	7
CHINA	INDIA	12	CHINA	ITALY	7
INDIA	AUSTRALIA	12	INDIA	CANADA	7

### 3.3 Affiliations performance

The core collection of literature on curcumin’s immune function pertains to 3500 research institutions globally. The top 20 institutions by publication volume are shown in the figure, with leading contributors such as the EGYPTIAN KNOWLEDGE BANK (EKB, 209 articles), MASHHAD UNIVERSITY OF MEDICAL SCIENCES (85 articles), ZAGAZIG UNIVERSITY (52 articles), Tehran University of Medical Sciences (51 articles), and the UNIVERSITY OF CALIFORNIA SYSTEM (50 articles). This reflects that over years of ongoing research, stable research teams and specialties have formed. However, the overall publication volume from institutions in this field is relatively low, suggesting a lack of continuity in research among most institutions. Although China ranks first in total publication volume, the top-ranking institutions are predominantly from other countries, with Shanghai Jiao Tong University being the highest-ranked institution in China at seventh place. Despite the relatively low individual publication output of Chinese institutions, nine institutions from China appear in the top 20, indicating widespread interest in curcumin among multiple Chinese research institutions, with a cumulative publication total of 367 articles, keeping China in the first position amongst research institutions.

**Figure 4:**
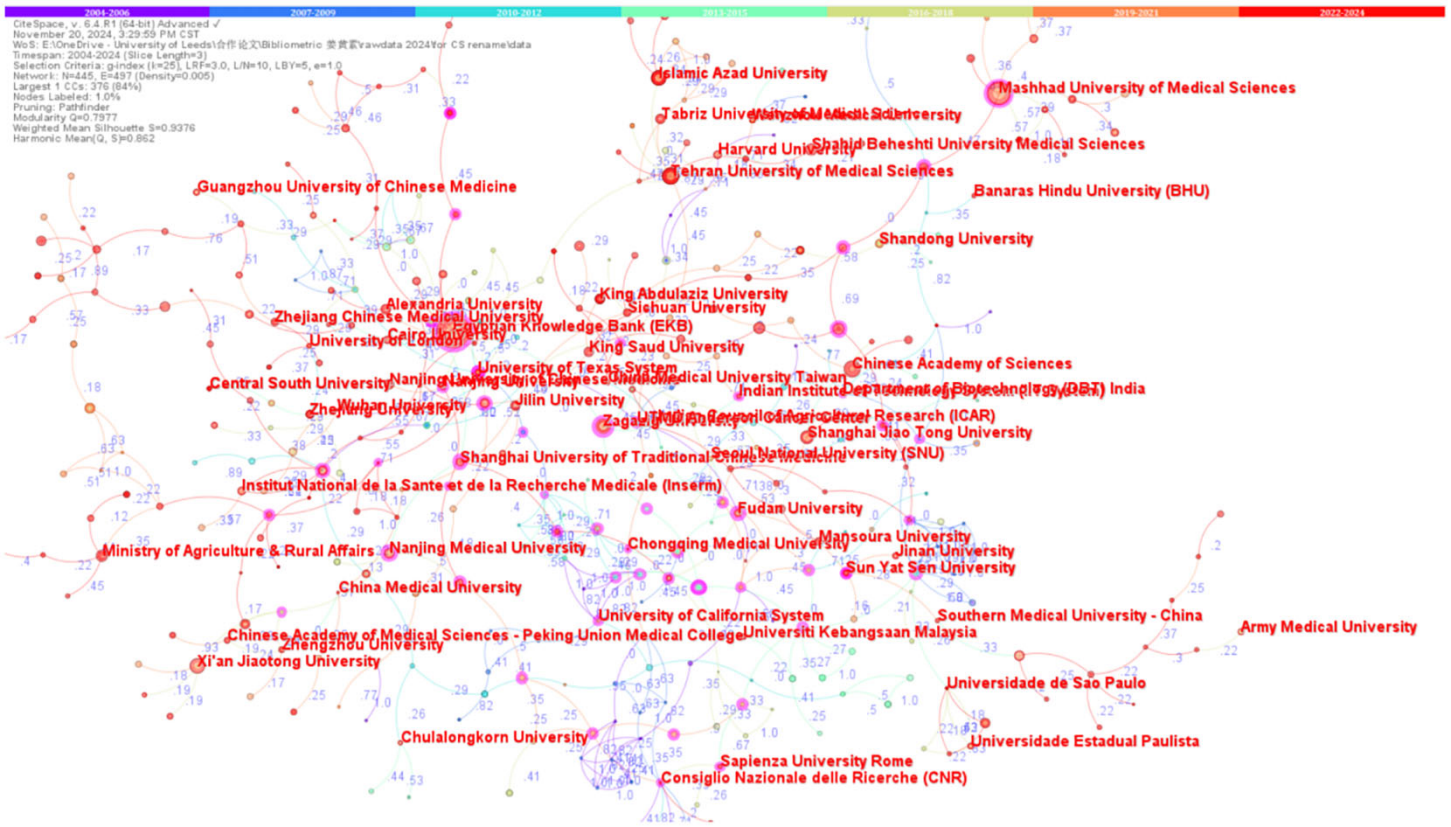
Research institutions’ cooperation network graph.

**Figure 4** depicts the collaboration network among institutions, where line thickness represents the frequency of cooperation and line color reflects the average year of those collaborations, with node and font size indicating the frequency of collaborations of specific institutions. Overall, there is a tight-knit academic cooperation circle among participants, achieving substantial outcomes in curcumin research. Over the past 20 years, cooperation between research institutions has intensified, with an increase in citation volume year by year. As shown in **Table 2**, Egyptian Knowledge Bank (EKB) has advantages in collaboration and citation volume. In recent years, Mashhad University of Medical Sciences and Tehran University of Medical Sciences have noticeably increased their collaborative research and citation output. However, the collaborations among institutions are still largely localized, with weak cross-regional partnerships and no significant establishment of an extensive cooperative relationship or academic community. Therefore, to shape future technological strategies in curcumin immune function research and promote resource exchange, sharing, and linking, more continuous interactions within the framework of leading forces in this field are needed.

**Table 2:** Research institutions’ publication volume.

#	Affiliations	Publications	% of 3939	Country	#	Affiliations	Publications	% of 3939	Country
1	EGYPTIAN KNOWLEDGE BANK EKB	209	5.306	Egypt	11	WENZHOU MEDICAL UNIVERSITY	41	1.041	China
2	MASHHAD UNIVERSITY OF MEDICAL SCIENCES	85	2.158	Iran	12	SICHUAN UNIVERSITY	40	1.015	China
3	ZAGAZIG UNIVERSITY	52	1.32	EGYPT	13	CAIRO UNIVERSITY	39	0.99	Egypt
4	TEHRAN UNIVERSITY OF MEDICAL SCIENCES	51	1.295	Iran	14	COUNCIL OF SCIENTIFIC INDUSTRIAL RESEARCH CSIR INDIA	39	0.99	India
5	UNIVERSITY OF TEXAS SYSTEM	50	1.269	USA	15	FUDAN UNIVERSITY	39	0.99	China
6	CHINESE ACADEMY OF SCIENCES	49	1.244	China	16	SUN YAT SEN UNIVERSITY	39	0.99	China
7	SHANGHAI JIAO TONG UNIVERSITY	49	1.244	China	17	XI AN JIAOTONG UNIVERSITY	36	0.914	China
8	ISLAMIC AZAD UNIVERSITY	47	1.193	Iran	18	INDIAN COUNCIL OF AGRICULTURAL RESEARCH ICAR	35	0.889	India
9	UNIVERSITY OF CALIFORNIA SYSTEM	45	1.142	USA	19	KING SAUD UNIVERSITY	34	0.863	Saudi Arabia
10	NANJING MEDICAL UNIV**ERSIT**Y	42	1.066	China	20	NANJING UNIVERSITY OF CHINESE MEDICINE	32	0.812	China

### 3.4 Journal performance and core author analysis

A review of the publications in the research sample database reveals that a total of 3939 articles are published across 1218 different journals, with 201 journals having published more than 5 articles, accounting for 59.24% of the total publication volume. As shown in **Table 3**, the top ten journals by publication volume include the *International Journal of Molecular Sciences*, which leads the field with 83 articles, representing 2.1% of all publications, followed by *Frontiers in Pharmacology*. Among the top 10 journals, the one with the highest impact factor is *Biomedicine Pharmacotherapy*.

**Table 3:** Journal publication volume.

Publication Titles	Local Impact				Global impact	
	Publications	H_index	Total Citations	Average Citations	JIF (2022)	JIF Quartile
International Journal of Molecular Sciences	83	26	2318	27.93	4.90	Q1
Frontiers in Pharmacology	61	21	1415	23.20	4.40	Q1
International Immunopharmacology	57	31	2761	48.44	4.80	Q1
Molecules	49	21	1687	34.43	4.20	Q2
Plos One	48	29	2832	59.00	2.90	Q1
Biomedicine Pharmacotherapy	47	20	1581	33.64	6.90	Q1
Frontiers in Immunology	38	15	985	25.92	5.70	Q1
Phytomedicine	37	20	996	26.92	6.70	Q1
Nutrients	35	19	1841	52.60	4.80	Q1
Scientific Reports	32	16	800	25.00	3.80	Q1

From the perspective of academic output, the top ten core authors are listed in **Table 4**. The researcher with the highest publication output in this field is Amirhossein Sahebkar from Mashhad University of Medical Sciences in Iran, with 56 publications. This institution ranks second in total publication volume. Other authors have significantly lower publication counts, highlighting the central position of Sahebkar in this field.

**Table 4:** Core authors’ publication volume.

Full Name	Publications	Affiliations	Country
Sahebkar, Amirhossein	56	Mashhad University of Medical Sciences	Iran
Jantan, Ibrahim	14	Taylor’s University	Malaysia
Liang, Guang	12	Wenzhou Medical University	China
Aggarwal, Bharat B	12	University of Texas M.D. Anderson Cancer Center	USA
Johnston, Thomas P.	11	University of Missouri Kansas City	USA
Kesharwani, Prashant	10	School of Pharmaceutical Education and Research, Jamia Hamdard, New Delhi	India
Patumraj, Suthiluk	10	Chulalongkorn University	Thailand
Majeed, Muhammed	10	Sabinsa Corp	USA
Bukhari, Syed Nasir Abbas	10	Jouf University	Saudi Arabia
Kong, Ah-Ng Tony	10	Shakibaei, Prof. Dr. Mehdi	USA

### 3.5 Highly cited papers

**Figure 5:**
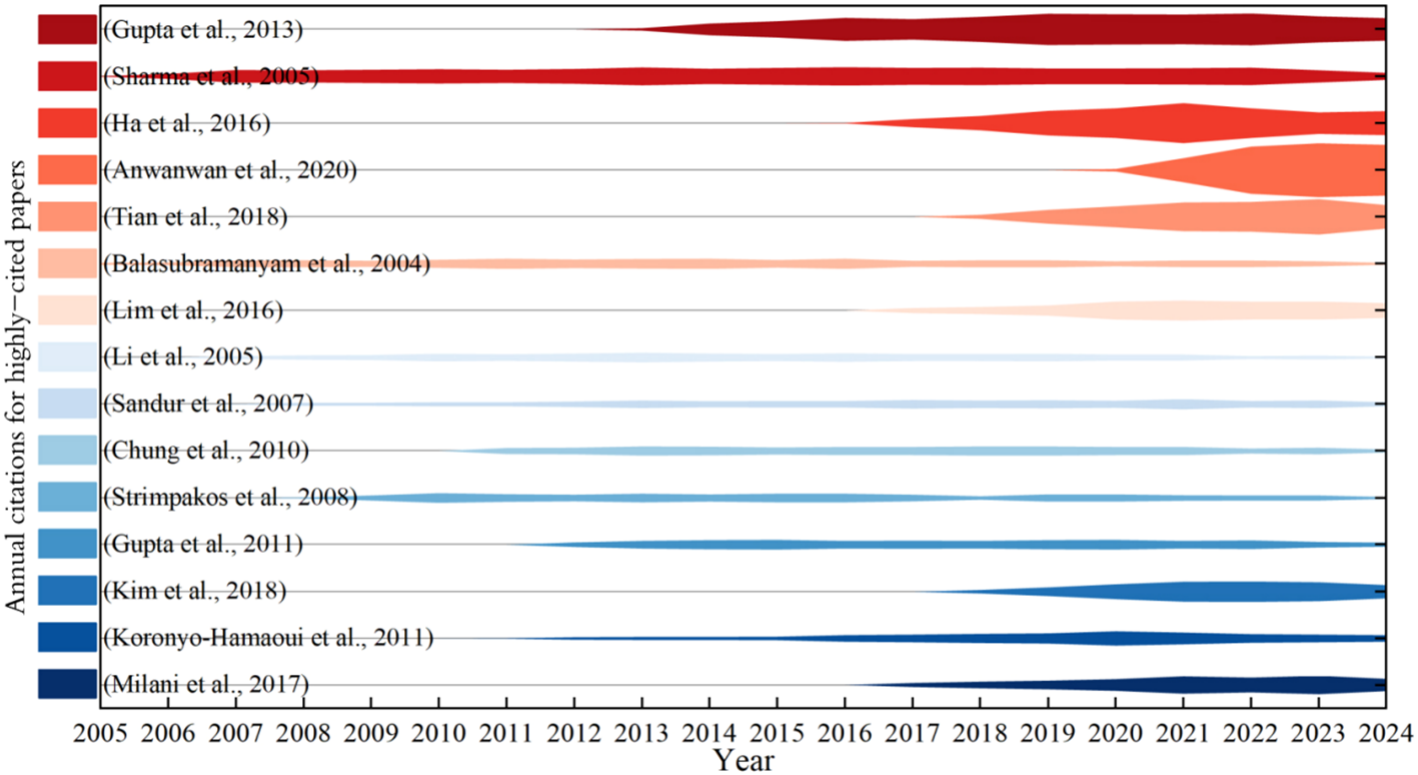
Citation trends of highly cited papers.

The volume of scientific literature produced is a reflection of the research development intensity in a specific area, while citation rates serve as a key metric for evaluating the impact of academic papers. In the **Table 5**,highly cited papers are considered foundational in their respective research fields and can be used to analyze the theoretical basis and methodologies of that area, reflecting the academic influence of the research body and the capacity to produce significant scientific results. As depicted in **Figure 5**, the width of the lines indicates varying citation volumes across different years. The most highly cited paper, (18) provides a comprehensive review of clinical trials involving curcumin in various human diseases, covering multiple fields such as cancer, inflammatory diseases, and diabetes. It analyzes the effects of curcumin as a monotherapy and its combination with other drugs, exploring various methods to enhance its bioavailability, thereby providing a systematic clinical basis. From 2013 to 2024, it has maintained a steady and high citation count, indicating its significant academic influence and reference value, and it lays the groundwork for subsequent large-scale, multi-center, long-term follow-up clinical trials. Other highly cited papers include (19–24), all reflecting stable citation counts from 2004 to 2024 without significant fluctuations. This indicates that research on the clinical therapeutic applications of curcumin is a focal point of study.In contrast, the high citation volume for papers like (18, 25–27) has notably increased post-2014, especially over the past three years, which reflects the growing interest in the therapeutic applications and mechanisms of curcumin.

**Table 5:** Highly cited papers.

#	Title	Citation	Total Citations	Average citations	Source Title
1	Therapeutic Roles of Curcumin: Lessons Learned from Clinical Trials	(18)	1334	111.17	AAPS JOURNAL
2	Curcumin: The story so far	(19)	1328	66.47	EUROPEAN JOURNAL OF CANCER
3	Exosomes as therapeutic drug carriers and delivery vehicles across biological membranes: current perspectives and future challenges	(25)	944	104.89	ACTA PHARMACEUTICA SINICA B
4	Challenges in liver cancer and possible treatment approaches	(27)	869	173.8	BIOCHIMICA ET BIOPHYSICA ACTA-REVIEWS ON CANCER
5	Surface functionalized exosomes as targeted drug delivery vehicles for cerebral ischemia therapy	(26)	776	110.86	BIOMATERIALS
6	Curcumin, a novel p300/CREB-binding protein-specific inhibitor of acetyltransferase, represses the acetylation of histone/nonhistone proteins and histone acetyltransferase-dependent chromatin transcription	(20)	648	30.86	JOURNAL OF BIOLOGICAL CHEMISTRY
7	Deubiquitination and Stabilization of PD-L1 by CSN5	(53)	550	61.11	CANCER CELL
8	Liposome-encapsulated curcumin - *In vitro* and *in vivo* effects on proliferation, apoptosis, signaling, and angiogenesis	(24)	535	26.75	CANCER
9	Curcumin,demethtoxycurcumin, bisdemethoxycurcumin, tetrahydrocurcumin and turmerones differentially regulate anti-inflammatory and anti-proliferative responses through a ROS-independent mechanism	(23)	528	29.33	CARCINOGE NESIS
10	Regulation of SIRT1 in cellular functions: Role of polyphenols	(22)	523	34.87	ARCHIVES OF BIOCHEMISTRY AND BIOPHYSICS

Analysis of the highly cited papers reveals that these works discuss significant scientific issues in the field of curcumin immune modulation from various perspectives and dimensions. Among them, five are original research articles while the remaining five are review articles, indicating that as related research deepens, researchers in the field seek to summarize and analyze previously published important methods, techniques, and conclusions. Hence, research innovation and summarization complement each other, driving academic advancement. Both original research articles and reviews have garnered substantial attention and high bibliometric value in the realm of curcumin immune function studies.

Curcumin exerts pharmacological effects through non covalent interactions with biomolecules (28). The pleiotropic effect of curcumin immunotherapy stems from its ability to regulate multiple signaling molecules by having multiple biological targets (6, 29). The potential therapeutic effects and mechanisms of curcumin in various diseases through the regulation of signaling molecules (30)or in synergy with them (31) is a primary focus of these highly cited papers, especially pertaining to synergistic cancer treatment (4, 32). The ten highly cited articles center around curcumin’s ability to regulate various signaling molecules, such as the role of exosomal curcumin complexes in drug delivery and the influence of curcumin liposomes on the NF-kB signaling pathway in pancreatic cancer.

### 3.6 References with the strongest bursts

**Figure 6:**
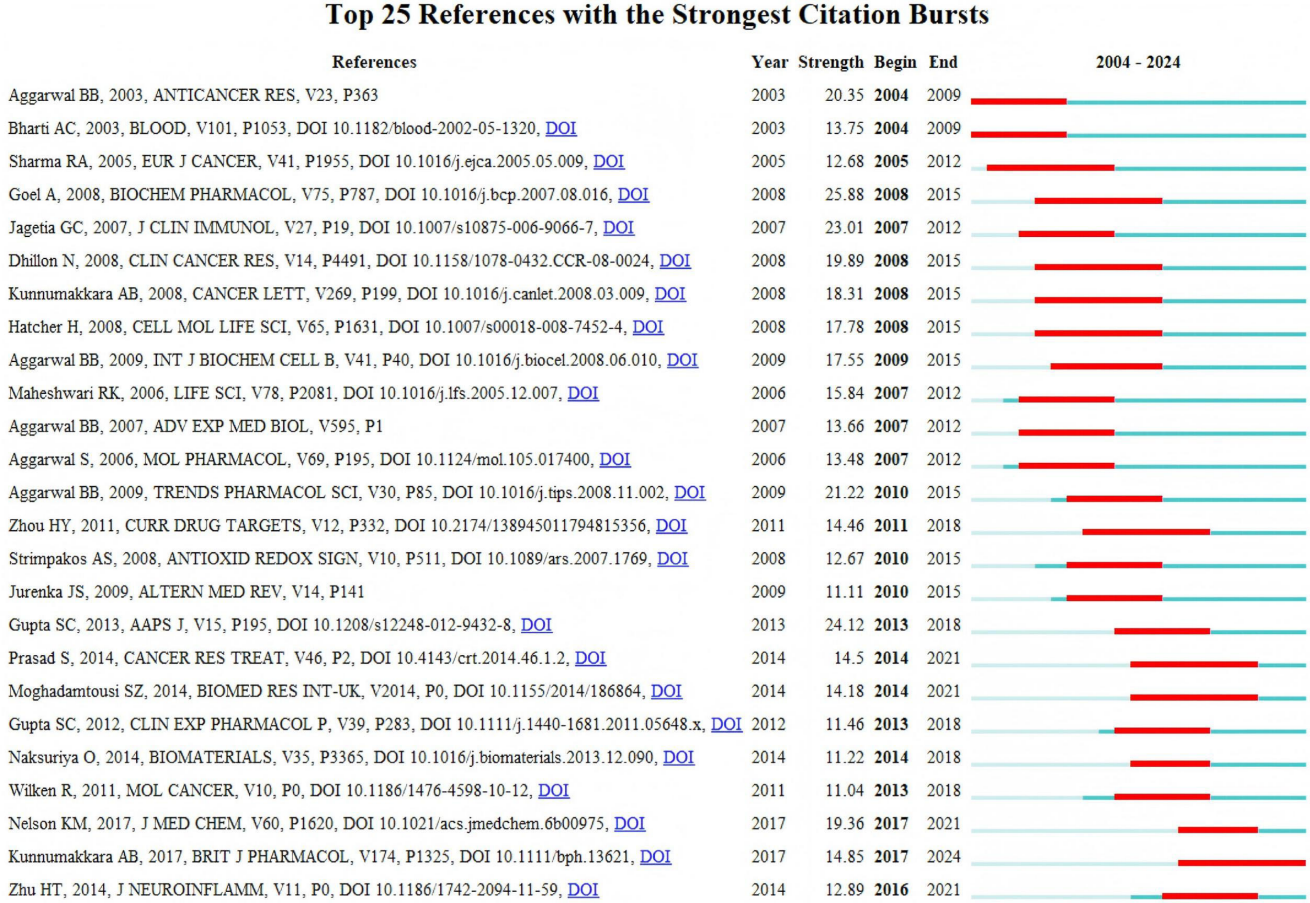
Active and inactive periods of reference citations.

The dynamics of research in a field can be partly characterized by articles that experience rapid increases in citation rates, known as citation bursts. **Figure 6** lists the 25 most frequently cited references (13, 18, 19, 21, 22, 33–52) for curcumin in the field of immune modulation. In the figure, red represents the active citation growth period, which is a time span during which a specific piece of literature or topic experiences rapid growth in citation counts and is frequently cited. During this period, researchers, scholars, and professionals show a high level of attention to the literature, often associated with new discoveries, emerging research hotspots, or advancements in related fields. Conversely, blue indicates the inactive state, referring to a time span during which the citation counts for a particular piece of literature, research topic, or field remain relatively stable, grow slowly, or decline. Identifying Active and Inactive periods in citation dynamics can help reveal the trajectory and forefront trends in academic research.A citation burst indicates potential attention from the scientific community regarding a given contribution. Additionally, references that begin to exhibit bursts are viewed collectively. As illustrated, seven references began to exhibit bursts in 2009, with the most notable one being Goel A. 2008 (34), published in Biochem Pharmacol, which summarizes half a century of the clinical potential of curcumin.

### 37 Co-citation networks

**Figure 7:**
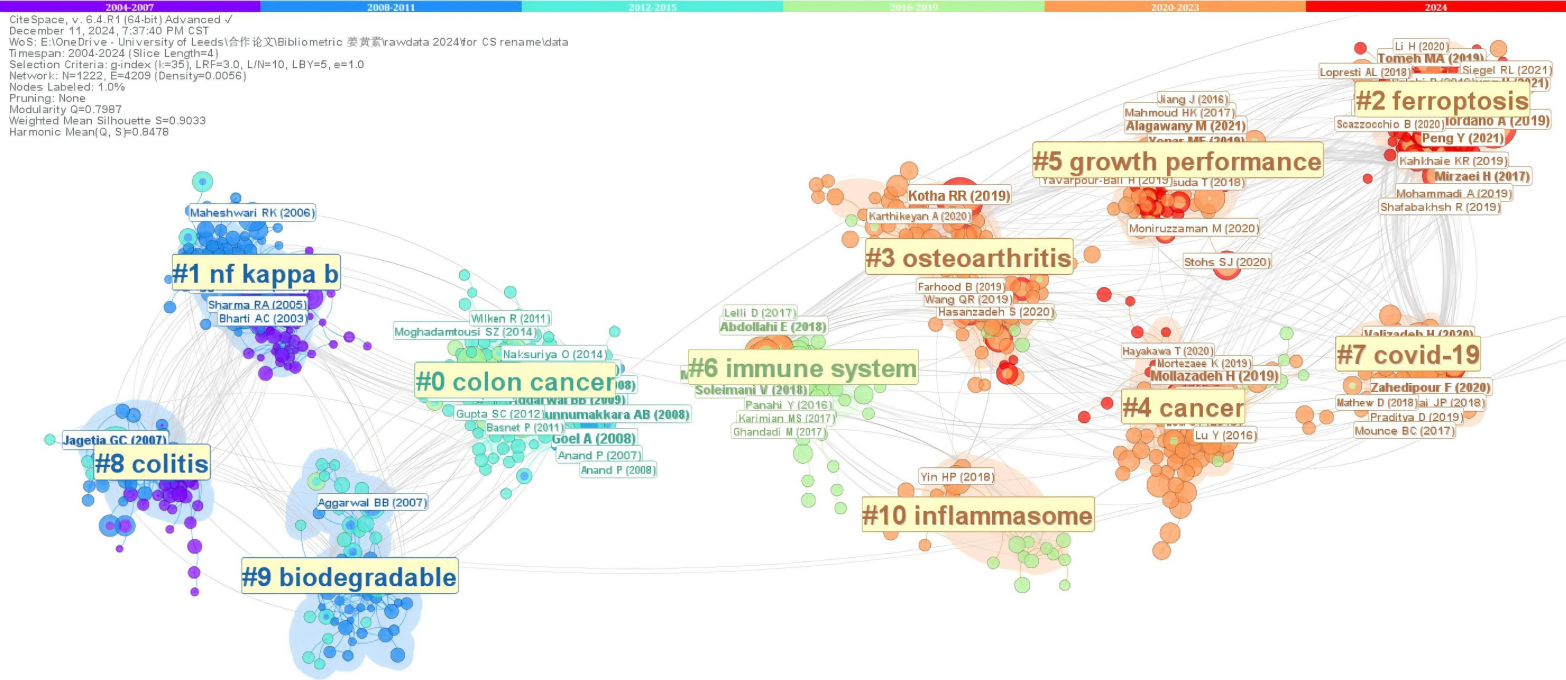
cluster view.

Using CiteSpace, we conducted a clustering analysis of highly cited references, showcasing the research hotspots and trends regarding curcumin from 2004 to 2024. Different colors of nodes and connections in the figure represent research focuses and relationships from different time periods. **Figure 7** shows ten clusters, including colon cancer, NF-kappa B, ferroptosis, osteoarthritis, cancer, growth performance, immune system, COVID-19, colitis, biodegradable, and inflammasome.

**Figure 8:**
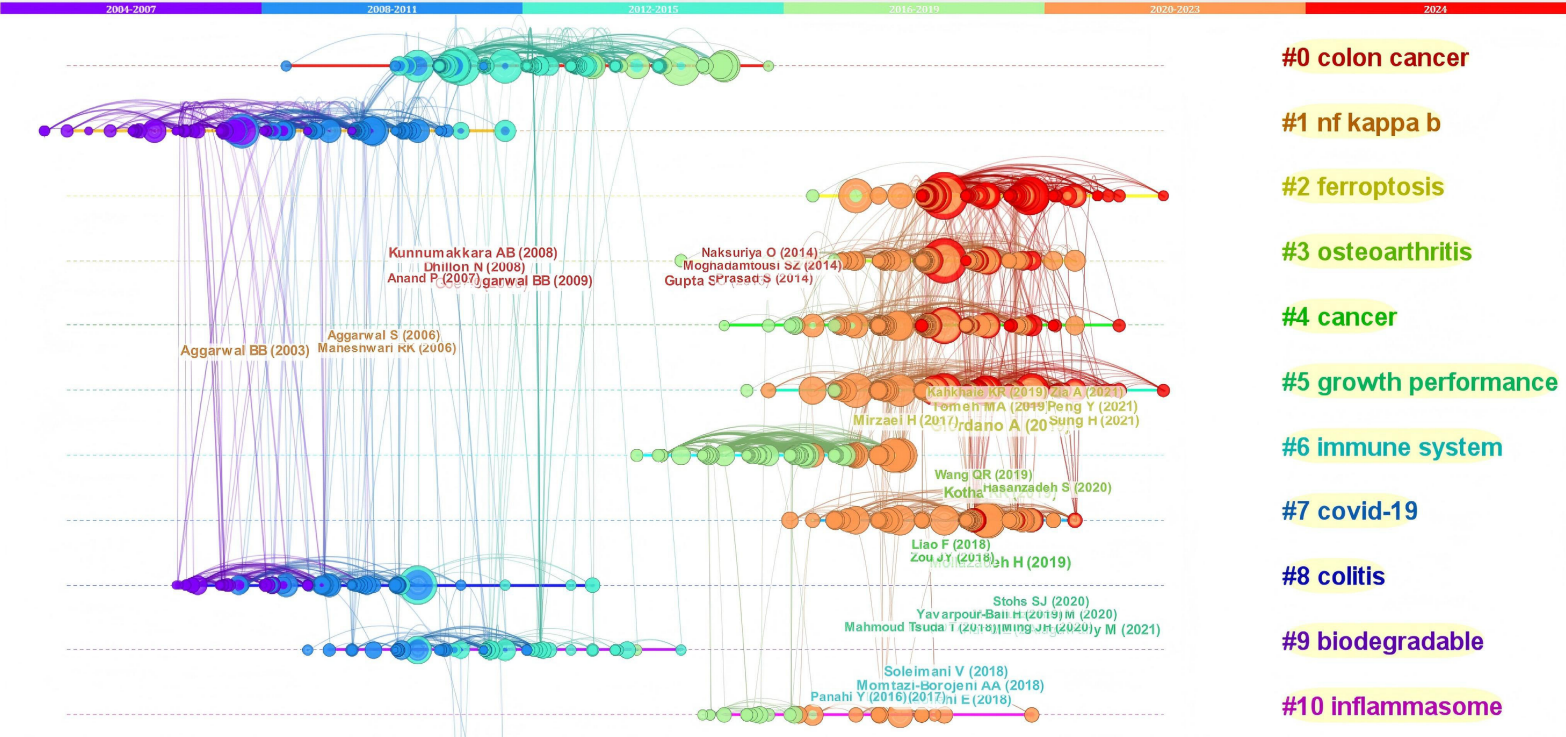
Timeline view.

**Figure 7** visualizes the trends in research across different time periods, showing a shift in research direction from pathology and biotechnology to broader health, environmental, and global challenges. Dividing the research landscape into two parts with 2015 as the cutoff, earlier studies concentrated on specific areas such as colitis and biodegradable substances, while later studies have expanded to health and environmental issues regarding COVID-19 and the emerging mechanisms of ferroptosis. Clusters #0 Colon Cancer and #8 Colitis indicate areas of focus that historically dominated immune research. Clusters #1 NF Kappa B and #9 Biodegradable are associated with immune responses and biodegradation in the **Figure 8**, indicating an ongoing focus on inflammation, immune mechanisms, and pharmacology.

From 2015 to the present, the research landscape has expanded and concentrated on ferroptosis, osteoarthritis, cancer, growth performance, immune system, COVID-19, and inflammasome. Clusters #2 Ferroptosis and #3 Osteoarthritis represent new areas of interest that particularly heightened after 2020, likely driven by aging and an increased focus on chronic diseases. Clusters #5 Growth Performance and #4 Cancer reflect a sustained interest and challenge within the biomedical domain. Cluster #7 COVID-19 indicates strong attention to the pandemic, especially post-2020, aligning with global pandemic response research needs. Clusters #6 Immune System and #10 Inflammasome involve ongoing in-depth exploration of immune mechanisms in the **Figure 8**.

Overall, the analysis indicates a gradual shift in research hotspots from basic pathology to broader social and environmental impact topics (e.g., COVID-19). Initial studies focused on specific diseases (e.g., colon cancer, osteoarthritis), whereas recent work has transitioned to encompass broader health and environmental concerns. The interconnections among research fields suggest that while clustering analysis categorizes articles, they are not limited to single themes but instead expand into a more comprehensive and multilayered research framework involving multiple themes.

### 3.8 Keywords

To investigate the influential research topics within a field over a period, we can analyze the explosive growth of keywords in that domain. A higher burst intensity for keywords within a certain timeframe indicates greater scholarly interest in the associated research themes, thus reflecting the significance of those themes during that period.

**Figure 9:**
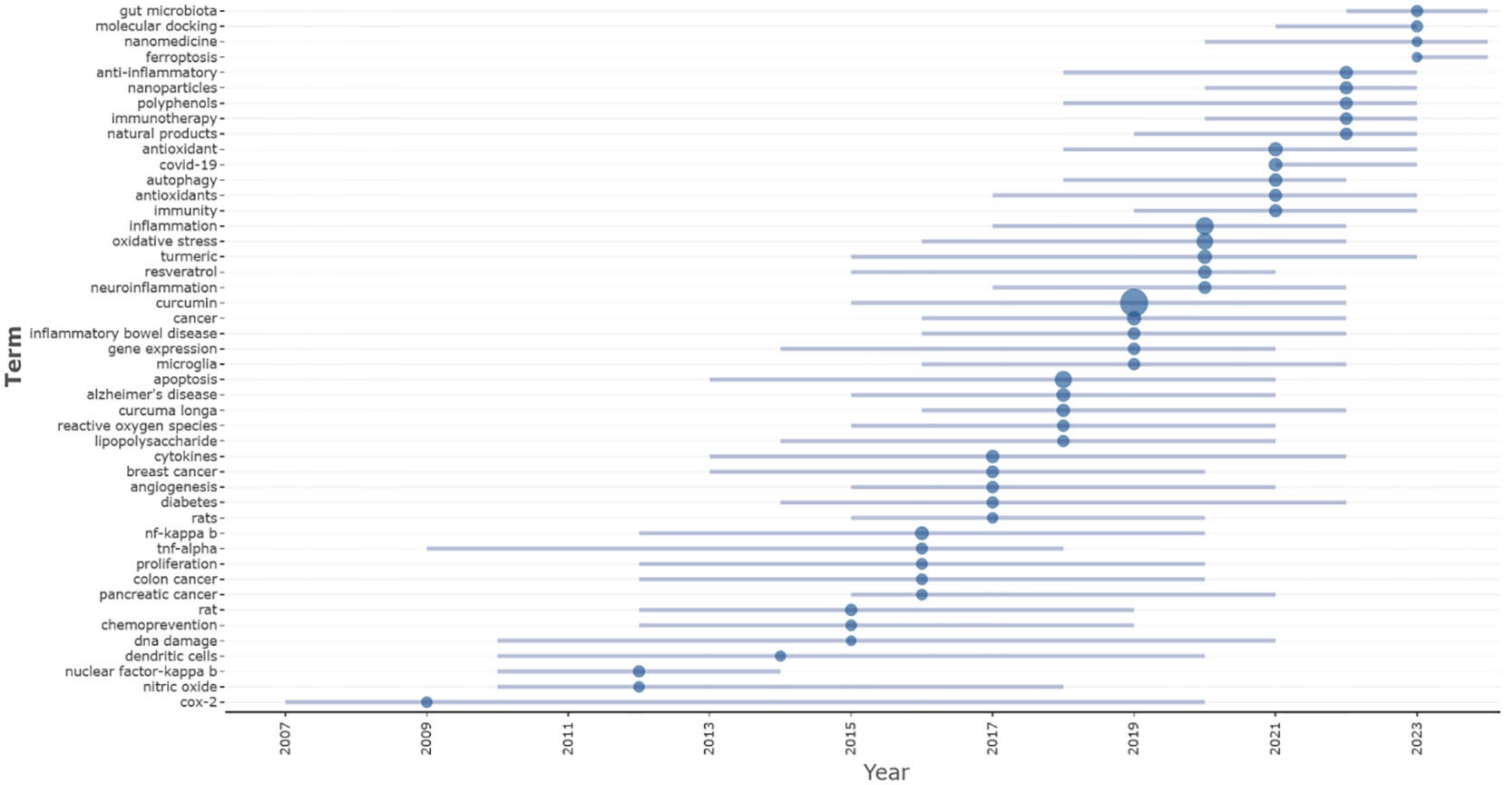
Keyword popularity evolution.

**Figure 9** displays an analysis of the temporal trends of research themes, utilizing R-bibliometrix to detect high-frequency keywords for each year, with a limit of at least 15 occurrences and a maximum of 5 keywords per year. The earliest explosive keyword, Cox-2, maintained a long duration of relevance from 2007 to 2019. Other early appearing keywords, such as DNA damage, TNF-alpha, and dendritic cells, show prolonged research popularity, indicating that early thematic hotspots were concentrated on molecular mechanisms, with sustained and deepening studies. As time progresses, the number of newly emerging keywords has increased, while the duration of their popularity has decreased, likely due to the continuous deepening of research and advancements in technology, leading to shorter research cycles and revolutionary progress. The size of the points in the figure represents citation volume. The top five keywords with the most explosive citations are curcumin (2014–2021), inflammation (2017–2022), apoptosis (2013–2021), oxidative stress (2016–2022), and cancer (2016–2022), revealing a deep investigation into their immunological effects within the contexts of inflammatory mechanisms, apoptosis, and cancer. Since 2021, newly emerged keywords with citation bursts include COVID-19, ferroptosis, and gut microbiota, indicating that curcumin research has become closely tied to the COVID-19 context in the past three years, explaining the high publication and citation volumes between 2020 and 2022.

### 3.9 Thematic evolution

**Figure 10:**
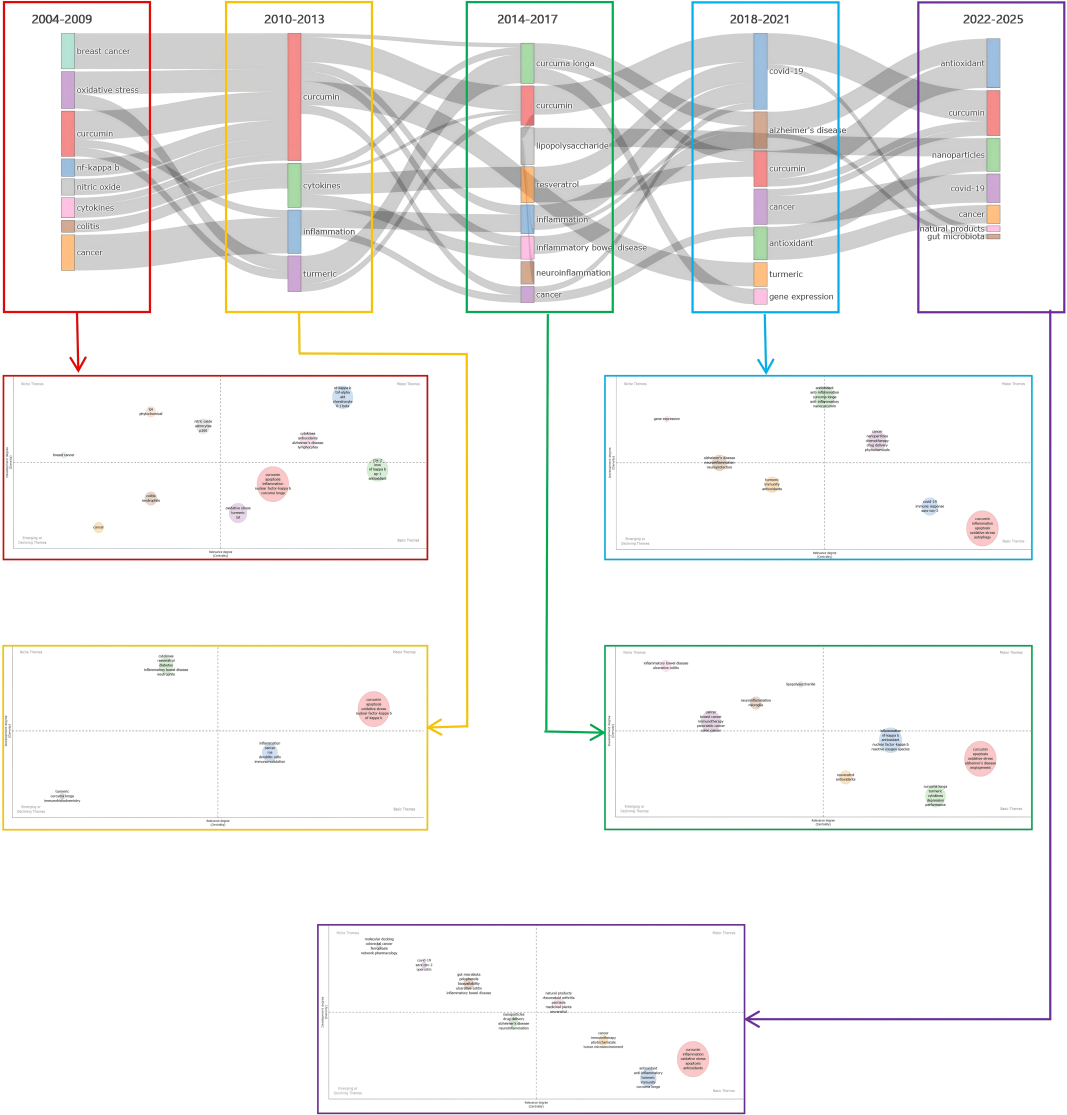
Temporal clustering strategicTables.

Utilizing R-bibliometrix, a strategic cluster diagram for five time zones has been drawn, as shown in the **Figure 10**. The horizontal axis represents centrality, indicating the degree of association with other themes; the vertical axis represents density, reflecting the interrelatedness of keywords within the field. The nodes represent the clustered themes. Nodes located in different quadrants represent different strategic significance: themes in the upper right quadrant are well-developed yet important, those in the lower right quadrant represent foundational but underdeveloped research themes, the upper left quadrant denotes stable professional themes, and the lower left quadrant represents emerging or declining themes.

Specific research themes for each time zone are elaborated here. Each five years is regarded as a sub-stage, where thematic associations are preserved by calculating the semantic similarity between technical themes, keeping only those associations exceeding a set threshold, with results visualized accordingly.

In the first sub-stage (2004–2009), eight major themes emerged. In the lower right quadrant, oxidative stress appeared as a prevalent theme, encompassing various application areas important to the immune regulation of curcumin, but it did not achieve further good development. In the lower left quadrant, colitis seems to be a declining theme, with low density and centrality, suggesting that the research may have matured and been absorbed into larger themes. In the upper left quadrant, breast cancer and nitric oxide are core components related to cancer treatment and biomedical research, showing good development in the field. In the upper right quadrant, NF-kappa B and cytokines are highly regarded, representing core themes of foundational research during this period.

In the second sub-stage (2010–2013), the research themes were dispersed across four major topics, with curcumin being a core hotspot in the upper right quadrant. The lower right quadrant contained one theme, inflammation, which exhibited lower central and density values, indicating weaker connections with other themes and the need for further development, while turmeric emerged in the lower left quadrant.

In the third sub-stage (2014–2017), the research themes evolved into eight, primarily concentrated in the upper left and lower right quadrants, with inflammatory bowel disease, cancer, neuroinflammation, and lipopolysaccharide residing in the upper left quadrant, having achieved substantial development. Curcumin, inflammation, resveratrol, and curcuma longa appeared in the lower right quadrant, closely related to antioxidant and anti-inflammatory research, thus gaining attention as potential research themes.

In the fourth sub-stage (2018–2021), research themes were organized into seven, distributed among all four quadrants. Cancer and antioxidant themes in the upper right quadrant are core hotspots, while curcumin in the lower right quadrant demonstrates a high degree of connection and potential for further development. The lower left quadrant includes a theme of turmeric that may represent an emerging topic.

In the fifth sub-stage (2022–2025), seven themes were concentrated in the right lower and left upper quadrants, indicating stable development in this sub-stage characterized by both stable and emerging themes. The COVID-19 theme in the left upper quadrant has developed steadily in alignment with the pandemic’s onset and treatment cycles. Themes in the right lower quadrant, including cancer, antioxidant, and curcumin, have good development potential. Emerging themes like nanoparticles and well-performing natural products in the lower left quadrant reflect a trend of technology integrating with nature, exhibiting strong growth potential.

A comprehensive analysis of the five time zones’ open access thematic strategic maps and clustering indicators reveals: (1) a wealth of relevant research theme keywords, with strong associations between early and later keywords establishing a foundation for subsequent research; (2) themes related to curcumin have continuously emerged as hotspots across the five time zones, with increasing centrality and density, intersecting with themes of anti-inflammation and antioxidants, deepening research continuously; (3) research themes tend to be distributed in the left upper and right lower quadrants, indicating that hotspot themes are developing steadily and a deepening of research is ongoing, with new hotspot keywords anticipated to develop further; (4) the evolution of research themes depicted in the figures not only reflects the changes in academic hotspots but also highlights the significant influence of external societal demands (such as pandemics). The anti-inflammatory effects of curcumin in various chronic inflammation-related diseases (such as autoimmune diseases, cardiovascular diseases, and metabolic syndrome) and its role in cancer prevention and treatment will continue to receive attention. Research will utilize multi-omics approaches, including genomics, proteomics, and metabolomics, to deeply investigate the mechanisms of action of curcumin, maintaining focus on specific mechanisms, potential clinical applications, and combination therapies targeting different inflammatory diseases and tumors. In light of the ongoing impact of the COVID-19 pandemic in recent years, investigating curcumin’s potential efficacy against other emerging viral diseases will also be a future hotspot. Additionally, research will place greater emphasis on curcumin’s effects on the gut microbiome, exploring its role as a natural product in promoting health, maintaining gut microbial balance, and its interactions with the immune system. Developing novel curcumin delivery systems, such as nanotechnology and carrier systems, to enhance its bioavailability and determine its clinical efficacy in various disease models will be a key focus.

## 4 Discussion

As a natural compound with clear immunopharmacological effects, curcumin has garnered significant research attention over the past twenty years. Bibliometric analysis of literature from 2004 to 2024 not only reveals the diverse effects of curcumin in immune modulation but also emphasizes its crucial roles in cancer, inflammation, and infectious disease treatments.

In terms of geographical distribution and collaboration trends, China dominates curcumin research, contributing 1433 publications, the highest globally. However, international cooperation remains relatively weak. Despite the USA’s lead in establishing extensive cooperative relationships and academic communities within the global curcumin research network, the cooperation between China and the USA, as well as other countries, remains limited. This lack of collaboration hampers the global expansion of research and resource sharing, indicating that strengthening international cooperation in the future will aid in integrating diverse scientific insights and resources. Furthermore, while numerous research institutions have made notable progress in related studies, large publication volumes are concentrated in a few countries such as Egypt, Iran, and China. Institutions like the EGYPTIAN KNOWLEDGE BANK (EKB) and Mashhad University of Medical Sciences exhibit excellent performance in terms of academic collaboration and literature impact but generally lack cross-regional collaboration. Although cooperative relationships among research institutions are becoming closer with an increase in citation volume, regional collaboration often impedes research globalization.

From bibliometric results, the hotspots and themes in curcumin research are continuously evolving. Early studies focused on specific diseases such as colon cancer and osteoarthritis, whereas recent hotspots have expanded to encompass broader health and environmental issues, such as COVID–19 and the emerging mechanism of ferroptosis. This expansion reflects scientists’ increasing attention to the potential applications of curcumin in modern medical challenges, especially its role in viral infections and immune modulation. In highly cited papers, we observe curcumin’s multifaceted ability to regulate various signaling molecules, particularly its synergistic effects in cancer treatment. In recent years, highly cited literature has increasingly focused on investigating the therapeutic uses and mechanisms of curcumin, indicating that as research deepens, scientists are becoming increasingly interested in the applications of this compound across various immune mechanisms.

## 5 Conclusion

Current research on curcumin is in a rapidly developing phase, with diversification and interdisciplinary applications continuously breaking new ground. With the acceleration of research into curcumin as a potential therapeutic agent due to the COVID-19 pandemic, the volume of literature and innovation in this field is expected to continue to grow. However, the explosive emergence of keyword hotspots and the surge in publication volume will still require specific timing. Additionally, current research collaborations are mostly limited to regional efforts, highlighting the need for increased international cooperation in the future. Through more effective global partnerships and innovative interdisciplinary research, curcumin’s therapeutic potential for immune-related diseases and emerging health challenges will be more comprehensively realized.

The research reveals that hotspots have shifted from specific diseases to broader health and social impact issues, indicating that the study of curcumin is not only deepening in the medical field but also playing a potentially key role in global health strategies. Researchers should continue to explore its molecular mechanisms and clinical applications in immune modulation to better address future health challenges.

## Data Availability

The original contributions presented in the study are included in the article/supplementary material. Further inquiries can be directed to the corresponding author.
